# Chemical Composition and Sensory Profiles of Fermented Cocoa Beans Obtained from Various Regions of Indonesia

**DOI:** 10.1155/2023/5639081

**Published:** 2023-03-11

**Authors:** Ariza Budi Tunjung Sari, Fahrurrozi, Tri Marwati, Titiek Farianti Djaafar, Retno Utami Hatmi, Purwaningsih, Yeyen Prestyaning Wanita, Puspita Lisdiyanti, Urip Perwitasari, Ario Betha Juanssilfero, Endang Sutriswati Rahayu

**Affiliations:** ^1^Indonesian Coffee and Cocoa Research Institute (ICCRI), Jember, 68118, Indonesia; ^2^Research Center for Marine and Land Bioindustry (RCMLB), National Research and Innovation Agency (NRIA), Jl. Raya Senggigi, Kodek Bay, Pemenang, Nort Lombok, West Nusa Tenggara 83352, Indonesia; ^3^Research Center for Food Technology and Process (RCFTP), National Research and Innovation Agency (NRIA), Yogyakarta, 55861, Indonesia; ^4^Asessment Institute for Agriculture Technology (AIAT) of Yogyakarta, Indonesia Ministry of Agriculture, Jl. Stadion Maguwoharjo No. 22, Ngemplak, Sleman, Yogyakarta, 55584, Indonesia; ^5^Research Center for Biosystematics and Evolution (RCBE), National Research and Innovation Agency (NRIA), Cibinong 16911, Indonesia; ^6^Research Center for Applied Microbiology (RCAM), National Research and Innovation Agency (NRIA), Jl. Raya Jakarta-Bogor Km 46, Cibinong, 16911, Indonesia; ^7^Centre for Food and Nutrition Studies, Gadjah Mada University, Yogyakarta 55281, Indonesia

## Abstract

The chemical composition and sensory profile of cocoa beans are essential factors determining the quality of cocoa-based products. In this study, cocoa bean samples were collected from various regions of Indonesia, including Aceh, Banten, Bali, East Java, West Sumatra, West Sulawesi, East Kalimantan, and Yogyakarta. The cocoa beans were fermented and sun-dried according to the producers' protocols and local practices. The sensory profile, fat content, total phenolic content, and the composition of sugars, organic acids, and amino acids of the cocoa bean samples were analyzed. The results revealed that the chemical composition and sensory profiles of the samples were diverse. The sensory profiles of cocoa liquor samples were described by low intensities of cocoa notes with the occurrence of fruity, floral, spicy, and sweet notes. The concentration of acetic acid, lactic acid, and some amino acids (glutamic acid, proline, and methionine) was associated with fresh fruit, browned fruit, and roasted note of the cocoa liquor, respectively. The variation in the environmental conditions and postharvest practices contributed to the diversity of cocoa beans' chemical and sensory characteristics.

## 1. Introduction

Cacao (*Theobroma cacao* L.) holds significant importance in the confectionery industry as the source of cocoa beans. Among several main producers of cocoa beans, Indonesia is one of the biggest in the world, with a production achieved to 734.800 tons/year [1]. In Indonesia, cacao is cultivated on all major islands with various agroecological conditions. This resulted in variations in cocoa beans' quality in the market (Febrianto and Zhu, [2]). Cocoa beans' quality is highly related to their chemical composition and sensory profile. Cocoa beans are mainly appreciated for their aroma and taste. The sensory profile of cocoa beans has a strong relationship with end-product quality, affecting consumer preferences for cocoa-based products [3].

Several factors affect the chemical composition and sensory properties of cocoa beans. These include the genetics of cocoa, growing environment (climate, soil, timing of sunlight, and rainfall), and postharvest processing (ripening, harvesting, fermentation, and drying) [2, 4–7]. These factors contribute to the diversity of chemicals, mainly flavors and their precursors in cocoa beans (Marseglia et al., [5]). Cocoa beans with diverse genotypes and origins have different flavor profiles (Kongor et al., [4]; Calva-Estrada et al., [7]). Rawel et al. [6] reported that the amino acid composition of cocoa beans changed during postharvest treatment. Febrianto and Zhu [2] found that the cocoa beans' phenolic and volatile composition was affected by the interaction of the environment and postharvest conditions. The study on the variations in the chemical composition and sensory quality of cocoa beans is important to better understand their relation to the origin and postharvest factors.

Most cocoa beans in Indonesia are unfermented. This produces cocoa products with intense bitterness and astringency taste but weak cocoa aroma. These characteristics are associated with low-quality cocoa beans. Fermentation is necessary to produce cocoa beans with good sensory characteristics (high-cocoa attributes). In smallholders, fermentation can be done using fresh cocoa beans at a minimum quantity of 5–40 kg. This is typically done using wooden boxes, bamboo baskets, or any convenient container [8]. Microbes such as yeast, lactic acid bacteria, acetic acid bacteria, and several genera of bacillus play crucial parts in spontaneous fermentation by secreting exogenous enzymes and secondary metabolites [9, 10]. A starter culture, such as a pure culture of *Lactiplantibacillus plantarum,* can be added to better control the fermentation [11] and prevent the growth of ochratoxin-producing *Aspergillus niger* [12–15]. A series of enzymatic activities by proteolytic, oxidase, and hydrolase enzymes induce the physicochemical changes in cocoa beans [16] such as the formation/reduction of phenolics, organic acids, amino acids, peptides, and reducing sugars as flavor precursors [4, 10, 17, 18]. These compounds give cocoa beans distinct flavor, taste, and color [19].

Attempts to produce fermented cocoa beans have been made in several producing regions of Indonesia. These areas have a different geographic and agricultural ecosystem, and postharvest practices. This may cause diversity in the chemical and sensory characteristics of fermented cocoa beans. The diversity of environmental conditions, cultivation methods, and postharvest practices in various parts of Indonesia is well represented in the characteristics of the 22 cocoa beans studied by Febrianto and Zhu [2]. However, sensory profiling on these beans has not been reported. Studies on the chemical and sensory characteristic of fermented Indonesian cocoa beans from different origins are scarce. In this study, eight samples of cocoa beans were collected from West Sulawesi, West Sumatra, Aceh, East Java, East Kalimantan, Bali, Banten, and Yogyakarta. The chemical and sensory profiles of the samples were analyzed. The results of this study are beneficial to better understand the relation of origin and postharvest practices to that of chemical composition and sensory profile. The results are also important for confectionery industries to seek cocoa raw materials with desired properties.

## 2. Materials and Methods

### 2.1. Materials

Cocoa bean samples were obtained from eight producing regions, including West Sulawesi, West Sumatra, Aceh, East Java, East Kalimantan, Bali, Banten, and Yogyakarta. Postharvest processing of cocoa beans was carried out by local practices; therefore, it should be noted that there were variations in the fermentation and drying protocols. Detailed information regarding the materials, geographical and agroecological conditions, and postharvest treatments are provided in Table 1. In the cocoa bean fermentation process, only those from Yogyakarta used the starter culture of *Lactobacillus plantarum* HL-15, while the fermentation of cocoa beans from other regions took place spontaneously. Dried fermented cocoa beans were packed in plastic bags and shipped to the Indonesian Coffee and Cocoa Research Institute (ICCRI). The samples were divided into smaller batches and distributed to the sensory laboratory of ICCRI, the laboratory of biotechnology of the Research Center for Biotechnology, the National Research and Innovation Agency (NRIA) in West Java, and the Assessment Institute for Agricultural Technology (AIAT) Yogyakarta for further analyses. The chemical used for extraction, standard, and analysis were of analytical and HPLC grade.

### 2.2. Chemical Characteristic Analysis

#### 2.2.1. Sample Preparation

The beans were peeled manually to separate the shell and the cotyledon. The cotyledons were then ground and sieved (#80 mesh). The resulting powder was then regarded as cocoa powder and stored in a sealed container until further analysis.

#### 2.2.2. Determination of Fat Content

The analysis of the total fat content of cocoa beans was performed according to AOAC 963.15. Five grams of cocoa powder was hydrolyzed using a solution of hydrochloric acid: water (2 : 1 v/v) for 15 minutes. The sample was then rinsed with water until AgNO_3_ drops showed a clear solution. The sample was dried for 16 hours before refluxing with n-hexane for 4 hours.

#### 2.2.3. Fermentation Index Analysis

The determination of fermentation indexes (FIes) was done following the method of [20] with modifications. A known weight (0.5 g) of cocoa powder was added to 50 mL of methanol (97 : 3 v/v) and incubated for 20 h. The mixture was then filtered and subjected to spectrophotometric readings at 460 nm and 530 nm (E-1000 V, Peak Instrument, Houston, TX). The fermentation index is the ratio of absorbance values at 460 nm to 530 nm. Well-fermented samples have FI value above 1, while a value below 1 indicates underfermentation.

#### 2.2.4. Determination of Total Phenolic Content

The total phenolic content was determined using the method of [21] based on Singleton and Rossi [22]. For the extraction of phenolics, cocoa powder was first defatted by Soxhlet extraction using hexane. Defatted cocoa powder (250 mg) was added with 40 mL of aqueous acetone (80%). The mixture was sonicated in an ice bath for 30 minutes and then vacuum-filtered. The filtrate was topped up with 80% aqueous acetone until the final volume of 50 mL. The filtrate was then subjected to TPC analysis. Filtrate (0.5 mL) was added with 2.5 mL of Folin-Ciocalteu reagent (0.2 N) and vigorously mixed. The solution was kept for 2 minutes at room temperature before adding 7.5 mL of saturated Na_2_CO_3_ solution. The solution was incubated for 2 hours in a dark condition. The blueish solution was then measured for absorbance at 765 nm using a spectrophotometer (E-1000 V, Peak Instrument, Houston, TX). A standard of catechin (Merck, Darmstadt, Germany) was used to develop a standard curve. The results were expressed as catechin equivalent per 100 g cocoa powder (dry matter basis, db).

#### 2.2.5. Quantification of Sugars, Organic Acids, and Ethanol

Two grams of cocoa powder were mixed with 6 mL of distilled water and homogenized in T25 Ultra Turrax (Staufen, Germany) for 5 min. The homogenate was centrifuged at 10,000 × *g* at 4°C for 15 min to obtain the supernatant. The extracts were filtered through 0.45-Minisart NY25 Syringe Filter (Sartorius, Gottingen, Germany) before being subjected to HPLC analysis. The concentrations of sugars (glucose and fructose), organic acids (citric acid, lactic acid, and acetic acid), as well as ethanol were quantified by high-performance liquid chromatography (HPLC) (LC-20AB, refractive index detector RID-10A, Shimadzu, Kyoto, Japan) equipped with an Aminex HPX-87H column (Bio-Rad, Richmond, CA) operated at a column temperature of 80°C. The mobile phase was 5.0 mM H2SO4 at a rate of 0.6 mL/min based on the method of Ardhana and Fleet [16] with modifications. The results were expressed as mg/g cocoa powder.

#### 2.2.6. Quantification of Amino Acids

Quantification of amino acids was carried out using the method of Adeyeye et al. [23] with modifications. The analysis consisted of several steps, such as hydrolysis step, derivatization, and followed by chromatography analysis. Hydrolysis was done on 0.050 gg defatted cocoa powder added with 5 mL HCl 6 N and purged with N_2_ for 5 minutes. The hydrolysis was done at 116°C for 24 hours in a vacuum condition. After hydrolysis, the tube was cooled, and the solution was filtered using Whatman paper no. 4. As much as 0.5 mL of solution was put into a 0.5 mL centrifuge tube and purged with N_2_ gas to dry. The powder was mixed with 3 mL of HCl (0.02 N) and vortexed until homogeneous. The solution was centrifuged at 3500 rpm for 15 minutes to obtain the supernatant. The supernatant was then filtered with a 0.45 mm Millipore filter. The same protocol was used to prepare sample, standard, and control solution. The samples were preheated in the oven at 55°C for 10 minutes prior to HPLC analysis. An HPLC system with Waters AccQ●Tag column (Alliance 2695 series, Waters, Milford, MA) was used to analyze the amino acids. HPLC conditions followed the method of Hinneh et al. [24]. The injection volume was 5 *μ*L. Mobile phases were buffer (AccQ●Tag) (a), Acetonitrile (b), and Milli Q water (c). The flow rate was 1 mL/min. Column's temperature was set to 37°C. The detection was done using a fluorescence detector set at the excitation wavelength of 250 nm and the emission wavelength of 395 nm. The standard solution used was 10 mL of amino acids standard dissolved in 20 mL of reagent powder AccQ2 and 10 mL of boric buffer. The results were expressed as mg/g cocoa powder free fat dry basis (ff db).

### 2.3. Sensory Evaluation

The sensory profile of cocoa beans was analyzed by a team of the panelist of the Cocoa of Excellence Program 2021 edition. The cocoa beans samples were sent to the International Center for Tropical Agriculture (CIAT) in Rome, Italy. The sample preparation and sensory attributes were performed according to International Standard for the Assessment of Cocoa Quality and Flavor [25]. The evaluation was performed by seven experts on various attributes, including cocoa, acidity, bitterness, astringency, fresh fruits, browned fruits, floral, woody, spicy, nutty, sweet/caramel, roasted, and off-flavors. The scoring criteria for intensities are comprised of 0–1 (not present—trace), 2–3 (weak), 4–5 (clear), 6–8 (strong), and 9–10 (overpowering). The scores of sensory attributes of cocoa beans from Ghana were used as a comparison. The use of the data has obtained permission from the committee of Cocoa of Excellence 2021 Edition and from the cocoa bean producers.

### 2.4. Statistical Anaysis

All the analyses were done in triplicate. The statistical analysis was carried out using SPSS software version 23 (IBM Corp., Armonk, NY, USA). Normality of distribution (Shapiro-Wilk's) and homogeneity of variance (Levene's) tests were performed prior to analysis of variance (ANOVA) and Tukey's honest significant difference post hoc tests. Correlational analysis was done on the sensory score and the concentration of chemical compounds, and Pearson's correlational coefficient (*r*) was determined. A 95% confidence level (*α* = 0.05) was applied in all tests. The sensory attributes of the cocoa beans were subjected for the principal component analysis (PCA) and the score plot was used to determine the possible grouping of the samples.

## 3. Results

### 3.1. Chemical Composition

#### 3.1.1. Fat and Fermentation Index

**Figure 1:**
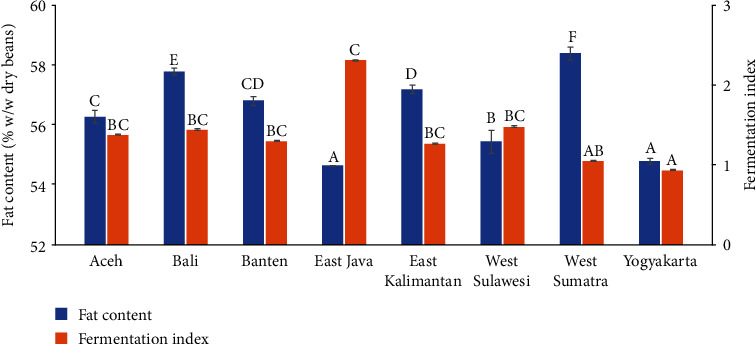
Fat content and fermentation indexes of cocoa beans samples from various regions of Indonesia. Bars represent standard deviation (*n = 3*). Bars with the same letter in the same group showed that the values were not significantly different (blue bars based on one-way ANOVA and Tukey's HSD tests, orange bars based on Kruskal-Wallis and Mann–Whitney's tests at 95% confidence interval).

The fat contents of Indonesian cocoa beans were in range of 54 to 58% (w/w db) (Figure 1). The fat content of East Java and Yogyakarta were the lowest, and West Sumatra was the highest. FIes of the samples were in the range of 0.9 to 2.3, indicating most of the beans have been fully fermented [26]. The lowest FI was found on the sample from Yogyakarta.

#### 3.1.2. Total Phenolic Content

**Figure 2:**
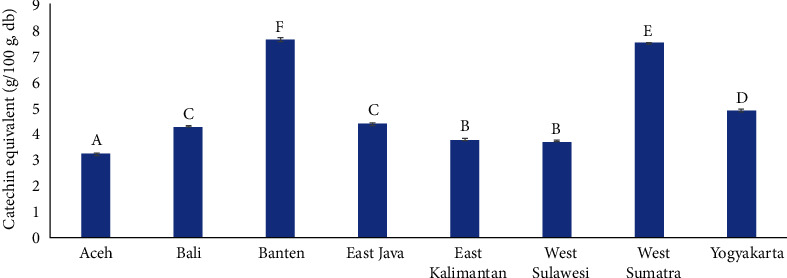
Total phenolic content of fermented cocoa bean from various regions in Indonesia. Bars with the same letter showed that the values were not significantly different (based on one-way ANOVA and Tukey's HSD tests at 95% confidence interval).

The total phenolic content of unroasted cocoa bean samples from Indonesia ranged from 3.2 to 7.6 g catechin equivalent/100 g cocoa powder. Cocoa beans from Banten had the highest TPC value, while Aceh had the lowest (Figure 2). This parameter was strongly correlated with astringency note (*r* = 0.898, *p* < 0.05). Samples with high TPC values showed strong astringency notes.

The cocoa beans originated from medium altitude (100–400 m.asl) regions such as Banten, West Sumatra, and Yogyakarta had higher levels of total phenolic content than those grown in altitudes of 0–100 m.asl, such as Aceh, Bali, and West Sulawesi.

#### 3.1.3. Sugars, Organic Acids, and Ethanol

**Figure 3:**
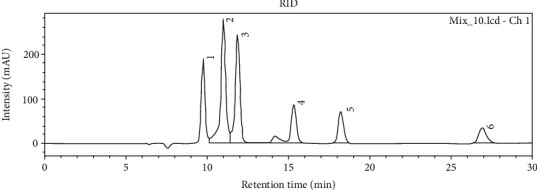
HPLC chromatogram of standards: (1) citric acid, (2) glucose, (3) fructose, (4) lactic acid, (5) acetic acid, and (6) ethanol.

**Figure 4:**
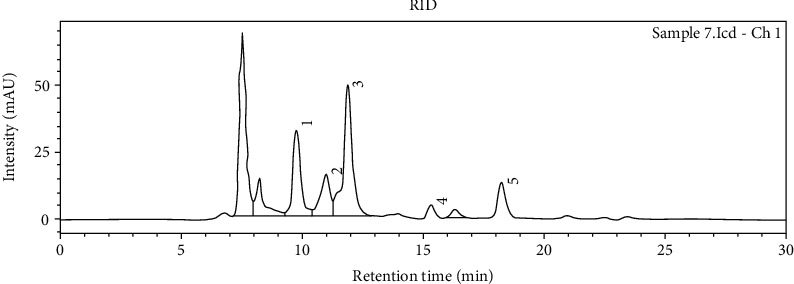
Representative HPLC chromatogram of cocoa bean sample from various regions in Indonesia.

The concentration of glucose and fructose in cocoa bean samples varied greatly. West Sulawesi sample had the lowest total concentration of glucose and fructose (0.125 and 0.735 mg/g, respectively). The highest glucose concentration (0.56 mg/g) and fructose (1.215 mg/g) was found in the sample from Banten (Table 2). Furthermore, the sample from Banten also had a higher concentration of lactic acid (0.435 mg/g) and a lower concentration of acetic acid (0.38 mg/g) than that of other regions. Inversely, cocoa beans from Yogyakarta had the highest concentration of acetic acid (1.335 mg/g) and the lowest concentration of lactic acid (0.135 mg/g). Ethanol was not detected in all samples of cocoa beans. HPLC chromatogram of standards and representative HPLC chromatogram of cocoa bean sample from various regions in Indonesia presented in Figures 3 and 4.

#### 3.1.4. Amino Acid

The cocoa bean samples from eight producing regions had a varied amino acid composition (Table 3). The total concentrations of free amino acids in cocoa bean samples were 58.30–86.18 mg/g cocoa powder ff db. This value was higher than that of Ghanaian cocoa beans (11.35–19.70 mg/g cocoa powder ff db) [24]. Cocoa beans from East Kalimantan had the highest total amino acid (86.18 mg/g cocoa powder db) and the hydrophobic free amino acid content (34.01 mg/g cocoa powder db). The ratios of hydrophobic to acidic AA were in the range of 1.03 to 1.29.

### 3.2. Sensory Characteristics

**Figure 5:**
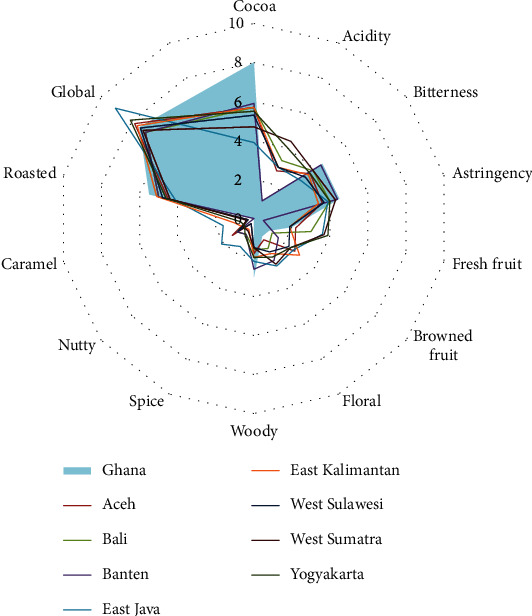
Sensory profile of cocoa bean samples from Ghana and various regions of Indonesia. The scores indicated intensity of each sensory attributes ranging from 0–1 (none to trace), 2–3 (weak), 4–6 (clear), 7–8 (strong), and 9–10 (overpowering). The values were mean of seven replications.

The sensory evaluation revealed that Indonesian cocoa beans are attributed with lower intensity of cocoa note (4.7 to 5.8) compared to that of Ghana (7.5) (Figure 5). The highest score of cocoa notes was found in cocoa beans from Banten, while the lowest was in the East Java beans. The acidity of Indonesian cocoa beans was generally stronger than Ghanaian beans, whose acidity score was 1. Cocoa beans from West Sumatra and Yogyakarta had the highest scores of acidities (4.4 and 4, respectively). The lowest acidity score was found in the sample from Banten (1). The bitterness and astringency observed in cocoa beans from Indonesia were slightly lower than that from Ghana (Figure 5).

Despite its low cocoa notes, cocoa beans from Indonesia were characterized by various additional notes. The samples from East Java, West Sumatra, and Yogyakarta demonstrated a clear sensation of fresh fruit and floral note. Samples from Aceh and East Kalimantan had browned fruit notes. The woody note defined in Ghanaian beans also found in Indonesian beans, particularly in the sample from Banten. The spicy note was present in all samples of Indonesian beans at a range of 1.5–2.6, but it was not detected in Ghanaian beans.

In terms of the overall profile, the beans from Banten had the closest profile to Ghana. It was characterized by a strong cocoa note and low acidity. However, cocoa beans from East Java had the highest global score at 9.1 despite their weak cocoa note. This was due to the flavor complexity of fresh fruit, spicy, and nutty notes. It also possessed the highest score for sweetness among other samples.

**Figure 6:**
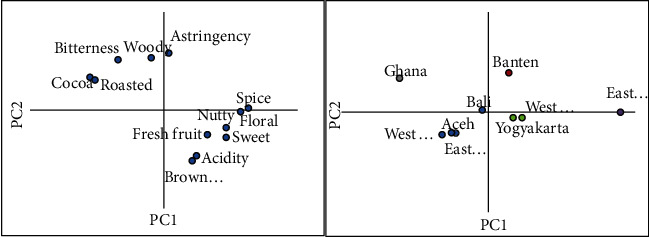
Loading plot (left) and scores plot (right) of principal component analysis on sensory attributes of cocoa bean from Ghana and various regions of Indonesia.

The PCA graph could better explain the variations of flavor profiles among the samples, whereas principal component (PC) 1 and 2 together explained 71.5% of total variances (PC1 55.9% and PC2 15.6%) (Figure 6). PCA results showed that Indonesian cocoa beans were classified into four groups. The first type was characterized by fresh and browned fruit notes, such as in the Yogyakarta and West Sulawesi samples. The second type had high intensity of spice, floral, and sweet notes, like in the East Java sample. The third was attributed with a woody note, exhibited in Banten cocoa beans. Lastly, the fourth group was characterized by a moderate intensity of all notes. This group consisted of cocoa beans from Aceh, Bali, East Kalimantan, and West Sumatra.

There was a positive correlation between the intensity of acidity with the concentrations of organic acids in cocoa beans. The acetic acid concentration strongly correlates with acidity (*r* = 0.820, *p* < 0.05) and fresh fruit notes (*r* = 0.868, *p* < 0.01). In contrast, the presence of citric acid was associated with a lower score of acidity (*r* = −0.713, *p* < 0.05). The concentrations of lactic acid were negatively correlated with acidity and fresh fruit notes, even though it does not reach statistical significance (*p* > 0.05). However, it was linked with browned fruit notes (*r* = 0.722, *p* < 0.05). The concentrations of most amino acids were positively linked with the roasted notes. These were observed mainly in glutamic acid (*r* = 0.751, *p* < 0.05), proline (*r* = 0.743, *p* < 0.05), and methionine (*r* = 0.769, *p* < 0.05). Detailed information on Pearson's coefficient of correlation was presented in Supplementary Table (available here).

## 4. Discussion

### 4.1. Chemical Composition

#### 4.1.1. Fat and Fermentation Index

The fat content is the most attractive feature of Indonesian cocoa beans. They are renowned for their hard cocoa butter with a high-melting point. Firmanto and Supriyadi [27] reported that the hardness of the cocoa butter was related to the rainfall (200–1000 mm/year), ambient temperature (26–29°C), and relative humidity (70–85%). Furthermore, it was suggested that the temperature of the environment had a positive correlation with the concentrations of palmitic acid, a significant component of saturated fatty acids in cocoa beans [28]. Interestingly, the fat content of cocoa beans from West Sumatra was significantly higher than others (*p* < 0.05), although environmental temperature was outside the range. The fat content might be associated with the genetic factor. Trinitario cocoa bean was reported to have higher fat content than that of Forastero [29].

The FI indicates the degree of fermentation based on the extent of polyphenol oxidation. Oxidized polyphenols have maximum absorbance at 460 nm, while the unoxidized ones are at 530 nm. FI near and above 1 indicates well-fermented beans, while unfermented and underfermented beans demonstrate a FI below 0.9 [30]. The FI was reported to be associated with the flavor quality of cocoa beans [26]. Currently, cocoa beans from Indonesia are sought after for their fat rather than their flavor. Hence, the beans can be sold regardless of the fermentation degree. The common practice among farmers is drying the cocoa beans under the sun directly after removing the husks. This resulted in cocoa beans produced by Indonesian farmers being mostly unfermented [31, 32].

#### 4.1.2. Total Phenolic Content

Phenolic compounds are abundant in cocoa beans. They are mainly from the flavonoid group. The predominant flavonoids are (−)-epicatechin, (+)-catechin, and their oligomer, namely procyanidins [33]. The phenolic compounds of cocoa beans exhibited various health benefits, such as for cardiovascular protection, anti-inflammatory, and antioxidant [34]. However, phenolic compounds are associated with undesired bitter and astringent tastes in cocoa products. Their presence lowered the intensity of cocoa notes in the cocoa liquor [35].

The total phenolic content decreased substantially in fermented beans (Febrianto and Zhu, [36]). During the fermentation, the phenolic compounds in the beans are oxidized by the polyphenol oxidase, forming brown-colored substances [37]. Previous studies reported that cocoa beans from Indonesia had high-phenolic content, resulting in strong bitterness and astringency [38]. Therefore, fermentation is essential to improve the sensory quality of cocoa beans.

Elevated levels of TPC value in cocoa beans grown at higher altitudes were also reported in another study [39]. In order to decrease the phenolic content, the exposure of phenolic compounds to the polyphenol oxidase shall be prolonged. Extending the duration of fermentation time could reduce the flavonoid and anthocyanin content in cocoa beans [2]. Furthermore, the phenolic compounds could be further reduced through drying at high temperatures in humid conditions [40].

#### 4.1.3. Sugar, Organic Acid, and Ethanol

The concentration of glucose and fructose content in cocoa beans from Banten was the highest compared to others. This was possibly due to limited microbial metabolism that utilizes fermentable sugar into organic acids in the beans during fermentation. This was confirmed by the low concentration of acetic acid in the beans. Microbes metabolize fermentable sugar into acetic acid, lactic acid, and ethanol [16]. In all samples, the concentrations of fructose were higher than glucose. This result was in agreement with a previous report of [41]. This was due to the hydrolysis of sucrose during the fermentation of cocoa beans; [42] found that some yeast preferred to metabolize glucose than fructose. High levels of glucose and fructose contributed to the taste of fresh fruit as found in cocoa beans from Banten.

Glucose and fructose from cocoa are the main carbon sources for yeast to metabolize ethanol [43]. However, in this study, ethanol was absent in all cocoa bean samples. During fermentation, ethanol produced by yeast could be metabolized into acetic acid by acetic acid bacteria [44]. Furthermore, the absence of ethanol might be related to the state of the samples. All the cocoa bean samples were previously dried for more than 4 days. This resulted in the complete evaporation of ethanol. Glucose was absent in the sample from Yogyakarta, possibly due to it was completely metabolized into acetic acid which was higher than other samples.

The concentration of sugars affects the quality of cocoa beans. Sugars are a substrate for microbes to metabolize organic acids, aldehydes, ketones, and other compounds. These compounds contributed to the flavor of cocoa beans [45]. During fermentation, yeast metabolizes citric acid and produces ethanol, pectinolytic enzymes, and volatile compounds. Lactic acid bacteria are the key to the production of lactic and citric acid. On the other hand, acetic acid bacteria are responsible for producing acetic acid through ethanol oxidation [43] However, despite their similar fermentation duration, cocoa beans from East Java, Bali, and Banten had different concentrations of sugar and organic acids. This might be due to the differences in the microbial diversity in each region. This was in agreement with the report of [37, 43].

When cocoa beans are heated, a reaction between reducing sugars and amino acids occurs, namely the Maillard nonenzymatic browning reaction [46]. This reaction affects the aroma of a product through the formation of volatile compounds such as furfurals and pyrazines. This reaction produces melanoidin, a compound that forms a brownish color in the product [47]. In this study, there was more fructose than glucose in the bean samples. Kim and Lee [48] found that the formation of melanoidins was faster in the fructose-amino acids system than in the glucose-amino acids system. However, the Maillard reaction involving these sugars could not produce furfural [49]. On the other hand, volatiles such as pyrazines were more influenced by amino acid sources, not sugar [46]. Indeed, the variations in sugars and amino acids in cocoa beans affect the formation of volatiles during roasting.

#### 4.1.4. Amino Acid

The genetic and environmental conditions of the sample's origin and postharvest practices affected the amino acid compositions of cocoa beans. There were three levels of free amino acid in cocoa beans, namely low (58–80 mg/g FFB), medium (80–140 mg/g FFB), and high (150–243 mg/g FFB) (Rohsius, Matissek, and Lieberei [50]). Cocoa beans from East Kalimantan had medium amounts of free amino acids. Other samples were classified as low. The sample from West Kalimantan was high-quality cacao beans. They had the total amino acids in the dried substances >80 mg/g [51–53]. Amino acids are precursors of Maillard reactions, along with reducing sugars. They are converted to the volatile component during cocoa beans roasting, generating pyrazines and aldehydes. These compounds give roasted, sweet, and chocolate notes to cocoa products. Ashoor and Zent [54] found that lysine and glycine were highly reactive during the Maillard reaction, while proline and methionine were at a moderate rate. Glutamic acid showed a low capability of producing brown color and pyrazines [55]. However, it induced the formation of 5-hydroxymethyl furfural from glucose and fructose [56]. The 5-hydroxymethyl furfural was important volatile in cocoa beans, contributing to caramel, waxy, and fatty notes (Li et al., [57]; Bonvehí, [58]).

In this study, the genotype of cocoa bean samples contributed to the differences in amino acid profile and concentration (Tables 1 and 3). This was in line with the study of Caligiani et al. [59] that the Criollo variety had higher levels of amino acids than that of Forastero and Trinitario. A previous study of Tran et al. [60] showed that well-fermented Criollo cocoa had a higher number of aroma precursors, which was further characterized by its fine flavor, compared to that of unfermented or partially-fermented, as well as well-fermented Forastero beans. Criollo had a high level of free total amino acids and a high ratio of hydrophobic to acidic amino acid. The ratio of hydrophobic to acidic amino acid in this study was higher (1.03–1.29) than that of Tran et al. [60](0.50). This indicated that the aroma of cocoa bean samples in this study was stronger. It was also supported by the study of Calva-Estrada et al. [7] that a high level of total amino acids and hydrophobic amino acids contributed to the strong cocoa aroma.

Fermentation induces changes in the total and hydrophobic amino acid levels in cocoa beans. Fermented cocoa beans had a higher total amount of free amino acids and hydrophobic amino acids than unfermented ones. Hydrophobic amino acids that increase during the fermentation were alanine, tyrosine, leucine, phenylalanine, valine, isoleucine, methionine, and proline (Rohsius, Matissek, and Lieberei [50]; Jinap et al., [61]; Sabahannur et al., [62]; Apriyanto, [53]; Hinneh et al., [24]; del Rosario Brunetto et al., [18]). The concentration of hydrophobic free amino acids fluctuated over fermentation duration [18]. The concentration of free hydrophobic amino acids (isoleucine, valine, leucine, alanine, gamma-aminobutyric acid, tyrosine, and phenylalanine) in underfermented cocoa beans (purple color) was higher than that of well-fermented (brown color) (Marseglia, Palla, and Caligiani, [63]). Jinap et al. [61] found that carboxypeptidase activity was high in underfermented cocoa beans (3 days of fermentation), resulting in a high concentration of total amino acids and hydrophobic amino acids. The concentrations were then decreased until the fermentation was complete (6 days) [61]. In this study, hydrophobic amino acid concentration increased along with fermentation duration (Tables 1 and 3). This might be attributed to environmental factors. These variations affected the performance of cocoa bean fermentation. For example, Calvo et al. [64] showed that low ambient temperature decreased the temperature in the fermentation system. This condition negatively impacted the formation of flavor and aroma precursors, resulting in cocoa beans with unfavorable aromatic and sensory profile.

In some samples of the cocoa beans, the cocoa pod was stored for several days before fermentation. According to Hinneh et al. [65], storage treatment of cocoa pods for up to 3 days did not affect the concentration of free amino acids, but storage for 7 days or more increased their concentrations in the beans. The drying treatment (cocoa bean thickness, drying time, and temperature) affected the concentration of free amino acids. Hashim et al. [66] found that drying in an 8.3 cm thick pile at 40°C produced beans with a high concentration of amino acids [66].

In this study, most cocoa bean samples were dried by sundrying for 4–5 days with an average drying temperature of 35–38°C. However, the piles' thickness varied from 1 to 3 cm of cocoa beans. Hence, these treatments might also contribute to the variations of free amino acid content in cocoa bean samples found in this study.

### 4.2. Sensory Profiles

Cocoa beans from Indonesia demonstrated a low intensity of cocoa note compared to that of Ghana. The cocoa note is contributed by pyrazines, a volatile product of the Maillard reaction [58]; Saltini, Akkerman, and Frosch, [67]). Despite their low intensity of cocoa notes, cocoa beans from Indonesia were rich in additional notes, particularly the fruity and floral notes. These notes were contributed by various volatile compounds, such as alcohols, aldehydes, ketones, esters, and terpenoids, and organic acids (citric, lactic, and acetic) (Rodriguez-Campos et al., [68]; Aprotosoaie, Luca, and Miron [69]). Esters, an important contributor of floral notes, are present in fresh beans and gradually diminish throughout fermentation but withstand high-temperature drying (60–80°C) [68]. Alcohols and acids increase as the fermentation progresses, mainly due to the metabolism of yeasts, lactic acid, and acetic acid bacteria. In the later stage of fermentation, the microorganisms' population declines along with the decrease of alcohols and acids [68]. These additional notes were an advantage of Indonesian cocoa beans over Ghanaian.

Genetics is an important factor affecting the sensory profiles of cocoa beans in this study. Forastero, Criollo, and Trinitario are the three main genetic types in cacao, in addition to other classifications such as Nacional. The Forastero cocoa beans are characterized by strong basic cocoa notes and are generally regarded as bulk or ordinary cocoa. Criollo cocoa has an aromatic flavor profile with nutty, earthy, and flowery notes. The Trinitario group (a hybrid of Criollo and Forastero) demonstrates a clear cocoa note with a wine-like aroma [69]. The cacao plants cultivated in Ghana are mostly Forastero hybrid, while Indonesian cacao plants are mostly a mix derived from the Upper Amazon Forastero, Trinitario, and Criollo genotypes (Kongor et al., [4]; Dinarti et al., [70]). Hence, it was reasonable for Indonesian cocoa beans to have a mix of sensory attributes but had lower cocoa notes than Ghanaians.

The sensory quality of cocoa beans is a major factor in determining the category of fine-flavor cocoa. The International Cocoa Organization has defined *fine cocoa* as cocoa beans with the complex flavor profiles and free from defects. This type of cocoa beans should also represent important genetic diversity and historical or cultural heritage [71]. *Fine cocoa* beans are mostly produced in Central and South American countries such as Brazil, Columbia, Costa Rica, and Dominica. They also include cocoa beans from Madagascar and Papua New Guinea [72]. There is no genetic specification of fine cocoa, even though mostly are constituted of Criollo and Trinitario, but other genetic such as Nacional also gains acknowledgement [69]. Indonesia is also regarded as one of the fine cocoa producers. The cocoa bean from East Java is the most recognized fine cocoa of Indonesia. It is a Criollo/Trinitario type and is being cultivated by estate-owned plantation, PT Perkebunan Nusantara XII [73].

PCA graph clearly showed that Indonesian cocoa bean samples in this study had sensory profiles more similar to *fine* East Java cocoa than *bulk* Ghanaians. This showed that Indonesian cocoa beans could be developed as *fine* cocoa. Indonesian cocoa beans have been acknowledged for their butter content but are less appreciated for their flavor qualities. Cocoa beans with good flavors are demanded by specialty chocolate makers and potentially sold at higher prices. Therefore, improving the flavor quality of cocoa beans is a strategy for farmers to achieve higher income. From this study, it was demonstrated that fermented cocoa beans exhibit desirable flavors. This could be a leverage for the image of Indonesian cocoa.

## 5. Conclusions

This study demonstrated multiple sensory profiles of fermented cocoa beans in Indonesia. The concentrations of the chemical compounds are attributed to the flavor variations. Genetics, geographical, climatic, and postharvest processing were among the influencing factors. Of these, modification of postharvest processing techniques is the most feasible means to modulate the flavor of cocoa beans. Finally, the diversity in the sensory characteristics allows the exploration of new sources of fine-flavor cocoa beans from Indonesia.

## Figures and Tables

**Table 1:** Detailed information of geographical conditions, genetic, and postharvest practices performed to obtain cocoa beans from various regions of Indonesia.

Indicators	Aceh	Bali	Banten	East Java	East Kalimantan	West Sulawesi	West Sumatra	Yogyakarta
*Geographic*								
Coordinate	96.2744 E 5.202861 S	-8.39206 E 144.83000 S	-6.497039 E 105.654765 S	7°14'06.1”S 112°44'00.8”E	1.84046529 E 117.24363519 S	3.4°S 119.3°E	0°48'49”S 100°37'34”E	110° 32' 16.7” E 7° 52' 23.7” S
Elevation (meters above sea level, m.asl)	0 – 100	0 – 100	100 – 400	0 – 100	0 – 100	0 – 100	100 – 400	100 – 400
*Climate*								
Temperature (°C)	30	26.34	27.9	28.04	26.93	27	22	26.65
Moisture (%)	80	81.00	90	70.00	85.67	94	97	82.17
Rainfall (mm)	674.67	201.00	215.75	156.18	149.21	158.27	181.15	193.92
*Genetic*								
Cocoa bean varieties	Forastero/Trinitario (local varieties)	Forastero	Forastero	Criollo	Forastero/Trinitario (local varieties)	Forastero (S-1, S-2, MCC-02 varieties)	Trinitario	Forastero
*Fermentation technique*								
Fermentation capacity (kg)	60	100	150	45	50	100	50	40
Duration in pod storage (days)	1	2	1	1	2	1	5	3
Duration of fermentation (days)	5	6	6	3	5	6	5	5
Number of reversals	3	2	3	2	3	3	1	2
Fermentation time (month)	November	June	May	October	October	September	December	July
*Drying technique*								
Drying time (month)	November	July	May	October	October	September	December	July
Length of drying (days)	5	5	10	10	5	4-5	4	5
Drying source	Sunlight	Sunlight	Sunlight	Sunlight	Sunlight	Solar dome	Sunlight	Sunlight
Drying facilities	Drying rack	—	Stainless steel drying rack	Drying floor	Drying rack	Greenhouse	Table with drying rack	Drying rack
Thick stack of cocoa beans (cm)	2	1–2	3	2	1–2	1–2	1–2	1–2

**Table 2:** The concentration of sugars, organic acids, and ethanol in cocoa bean samples from various regions of Indonesia.

Parameter (mg/g)	Origin							
	West Sulawesi	West Sumatra	Aceh	East Java	East Kalimantan	Bali	Banten	Yogyakarta
Glucose	0.13 ± 0.00	0.35 ± 0.01	0.23 ± 0.02	0.26 ± 0.01	0.37 ± 0.02	0.22 ± 0.00	0.56 ± 0.01	0.00 ± 0.00
Fructose	0.74 ± 0.00	0.98 ± 0.00	0.88 ± 0.04	1.08 ± 0.01	1.17 ± 0.02	1.05 ± 0.02	1.22 ± 0.02	1.16 ± 0.01
Lactic acid	0.22 ± 0.01	0.21 ± 0.00	0.27 ± 0.00	0.25 ± 0.00	0.17 ± 0.01	0.37 ± 0.00	0.44 ± 0.00	0.14 ± 0.00
Acetic acid	0.39 ± 0.00	1.29 ± 0.02	0.67 ± 0.00	0.97 ± 0.02	0.91 ± 0.02	0.80 ± 0.00	0.38 ± 0.00	1.34 ± 0.02
Ethanol	n.d.	n.d.	n.d.	n.d.	n.d.	n.d.	n.d.	n.d.

^∗^n.d., not detected.

**Table 3:** Fermented cocoa bean amino acid profile from various regions in Indonesia.

Amino acid profile (mg/g)	Origin								SD
	West Sulawesi	West Sumatra	Aceh	East Java	East Kalimantan	Bali	Banten	Yogyakarta	
Hydrophobic									
Alanine	4.45	1.25	1.27	1.63	4.11	1.49	4.72	1.58	0.01
Tyrosin	2.76	2.70	3.29	2.74	2.53	2.49	3.18	3.12	0.01
Valine	3.91	3.90	3.84	3.81	3.60	4.21	4.72	4.60	0.14
Isoleucine	2.38	2.41	2.30	2.36	2.22	2.85	2.89	2.81	0.09
Leucine	4.04	4.15	4.01	3.99	3.66	4.51	5.02	4.89	0.15
Phenylalanine	3.23	3.37	4.09	3.07	2.92	3.26	4.25	3.77	0.12
Methionine	1.06	0.81	0.99	0.98	0.85	0.74	1.07	0.98	0.04
Proline	5.39	5.00	5.02	6.09	4.68	4.74	8.16	5.50	0.16
Total	27.22	23.59	24.81	24.67	24.57	24.29	34.01	27.25	0.72
Acidic									
Aspartic acid	6.71	6.25	5.30	6.73	6.02	7.02	7.91	7.92	0.23
Glutamic acid	11.26	10.93	9.23	10.85	10.28	10.46	13.23	13.26	0.37
Histidine	1.23	1.28	1.54	1.14	1.22	1.18	1.55	1.40	0.04
Serin	3.24	3.32	3.69	3.17	3.11	3.21	3.77	3.87	0.12
Total	22.44	21.78	19.76	21.89	20.63	21.87	26.46	26.45	0.76
Others									
Glysine	2.74	3.28	3.07	2.66	2.70	3.09	7.75	3.49	0.07
Arginine	4.17	4.87	5.34	4.67	7.01	4.53	6.37	5.71	0.16
Treonine	2.76	2.69	2.99	2.81	2.52	3.11	3.08	3.44	0.10
Lysine	9.18	1.67	1.42	2.56	8.23	1.93	7.56	2.36	0.06
Cysteine	0.84	0.70	0.91	0.81	0.78	0.58	0.95	0.76	0.02
Total	19.69	13.21	13.73	13.51	21.24	13.24	25.71	15.76	0.41
Grand total	69.35	58.30	66.44	59.40	86.18	60.07	69.46	58.58	
Hydrophobic AA/acidic AA	1.21	1.08	1.26	1.13	1.19	1.11	1.29	1.03	

^∗^SD, standard deviation.

## Data Availability

The data presented in this study are available on request from the corresponding author.

## Supplementary Materials

Supplementary Materials Supplementary Table 1: Pearson's coefficient of correlations of the sensory attributes and chemical compounds. Values with ^∗^ and ^∗∗^ were significant at confidence level 95% and 99%, respectively. Supplementary Table 2: Pearson's coefficient of correlations of amino acid content and the other chemical compounds. Values with ^∗^ and ^∗∗^ were significant at confidence level 95% and 99%, respectively. Supplementary Table 3: Pearson's coefficient of correlations of organic acid, sugars, fat, and total phenolic content and fermentation index. Values with ^∗^ and ^∗∗^ were significant at confidence level 95% and 99%, respectively.
