# Supplementary material for: Chemical Composition and Sensory Profiles of Fermented Cocoa Beans Obtained from Various Regions of Indonesia
Source: Int J Food Sci. 2023 Mar 11;2023:5639081. doi: 10.1155/2023/5639081 (PMC10024629; doi:10.1155/2023/5639081)
Supplement: Supplementary Materials — Supplementary Table 1: Pearson's coefficient of correlations of the sensory attributes and chemical compounds. Values with ∗ and ∗∗ were significant at confidence level 95% and 99%, respectively. Supplementary Table 2: Pearson's coefficient of correlations of amino acid content and the other chemical compounds. Values with ∗ and ∗∗ were significant at confidence level 95% and 99%, respectively. Supplementary Table 3: Pearson's coefficient of correlations of organic acid, sugars, fat, and total phenolic content and fermentation index. Values with ∗ and ∗∗ were significant at confidence level 95% and 99%, respectively. [file 5639081.f1.docx]

Suplementary Table 1. Pearson’s coefficient of correlations of the sensory attributes and chemical compounds. Values with * and ** were significant at confidence level 95% and 99%, respectively.

|  | Cocoa | Acidity | Bitterness | Astringency | Fresh fruit | Browned fruit | Floral | Woody | Spice | Nutty | Sweet | Roasted | Global |
| --- | --- | --- | --- | --- | --- | --- | --- | --- | --- | --- | --- | --- | --- |
| Cocoa | 1 | -0.363 | 0.650 | -0.124 | -0.602 | -0.314 | -0.638 | 0.077 | -0.600 | -0.563 | -0.702 | .837^**^ | -0.692 |
| Acidity | -0.363 | 1 | -0.354 | -0.063 | .882^**^ | 0.260 | 0.091 | -.710^*^ | 0.010 | -0.347 | 0.252 | -0.389 | 0.141 |
| Bitterness | 0.650 | -0.354 | 1 | 0.613 | -0.474 | -0.590 | -0.044 | 0.273 | -0.211 | -0.465 | -.777^*^ | 0.536 | -.771^*^ |
| Astringency | -0.124 | -0.063 | 0.613 | 1 | 0.061 | -0.623 | 0.576 | 0.253 | 0.389 | -0.068 | -0.367 | -0.131 | -0.284 |
| Fresh fruit | -0.602 | .882^**^ | -0.474 | 0.061 | 1 | 0.189 | 0.260 | -0.431 | 0.424 | 0.035 | 0.500 | -0.645 | 0.482 |
| Browned fruit | -0.314 | 0.260 | -0.590 | -0.623 | 0.189 | 1 | 0.018 | -0.044 | -0.156 | 0.023 | 0.563 | -0.390 | 0.545 |
| Floral | -0.638 | 0.091 | -0.044 | 0.576 | 0.260 | 0.018 | 1 | 0.361 | 0.687 | 0.082 | 0.414 | -0.356 | 0.289 |
| Woody | 0.077 | -.710^*^ | 0.273 | 0.253 | -0.431 | -0.044 | 0.361 | 1 | 0.505 | 0.291 | 0.183 | 0.075 | 0.271 |
| Spice | -0.600 | 0.010 | -0.211 | 0.389 | 0.424 | -0.156 | 0.687 | 0.505 | 1 | 0.498 | 0.639 | -0.429 | 0.611 |
| Nutty | -0.563 | -0.347 | -0.465 | -0.068 | 0.035 | 0.023 | 0.082 | 0.291 | 0.498 | 1 | 0.461 | -0.556 | 0.638 |
| Sweet | -0.702 | 0.252 | -.777^*^ | -0.367 | 0.500 | 0.563 | 0.414 | 0.183 | 0.639 | 0.461 | 1 | -0.507 | .873^**^ |
| Roasted | .837^**^ | -0.389 | 0.536 | -0.131 | -0.645 | -0.390 | -0.356 | 0.075 | -0.429 | -0.556 | -0.507 | 1 | -.725^*^ |
| Global | -0.692 | 0.141 | -.771^*^ | -0.284 | 0.482 | 0.545 | 0.289 | 0.271 | 0.611 | 0.638 | .873^**^ | -.725^*^ | 1 |
| Citric Acid | -0.077 | -.713^*^ | 0.219 | 0.415 | -0.369 | -0.292 | 0.469 | .926^**^ | 0.651 | 0.446 | 0.170 | 0.013 | 0.268 |
| Acetic acid | -0.434 | .820^*^ | -0.207 | 0.126 | .868^**^ | 0.330 | 0.381 | -0.203 | 0.391 | -0.230 | 0.470 | -0.486 | 0.362 |
| Lactic acid | 0.435 | -0.627 | 0.610 | 0.295 | -0.576 | -.722^*^ | -0.309 | 0.210 | -0.079 | 0.233 | -0.548 | 0.441 | -0.534 |
| Glucose | 0.022 | -0.553 | 0.513 | 0.380 | -0.538 | -0.120 | 0.403 | 0.529 | 0.137 | 0.088 | -0.110 | 0.210 | -0.280 |
| Fructose | 0.072 | -0.139 | 0.308 | 0.253 | 0.037 | 0.024 | 0.445 | .720^*^ | 0.581 | -0.126 | 0.328 | 0.125 | 0.193 |
| Aspartic | 0.298 | -.744^*^ | 0.108 | -0.185 | -0.661 | -0.099 | 0.101 | 0.688 | 0.202 | 0.122 | 0.161 | 0.580 | 0.001 |
| Serine | 0.225 | -0.288 | 0.592 | 0.352 | -0.438 | -0.004 | 0.470 | 0.433 | -0.008 | -0.455 | -0.170 | 0.427 | -0.425 |
| Glutamic | 0.555 | -.727^*^ | 0.317 | -0.167 | -.773^*^ | -0.092 | -0.022 | 0.629 | -0.058 | -0.172 | -0.091 | .751^*^ | -0.235 |
| Glycine | 0.226 | -0.089 | -0.044 | -0.494 | -0.251 | 0.479 | 0.054 | 0.237 | -0.088 | -0.342 | 0.345 | 0.466 | -0.011 |
| Histidine | 0.098 | 0.032 | 0.278 | 0.021 | -0.190 | 0.437 | 0.385 | 0.217 | -0.135 | -0.540 | 0.069 | 0.241 | -0.208 |
| Arginine | 0.469 | -0.263 | 0.328 | -0.321 | -0.386 | 0.386 | -0.466 | 0.216 | -0.437 | -0.125 | -0.181 | 0.191 | -0.159 |
| Threonine | -0.150 | -0.503 | 0.314 | 0.444 | -0.410 | -0.176 | .717^*^ | 0.638 | 0.435 | 0.075 | 0.124 | 0.208 | -0.088 |
| Alanine | 0.340 | -0.196 | -0.307 | -.847^**^ | -0.465 | 0.509 | -0.608 | -0.251 | -.726^*^ | -0.097 | -0.046 | 0.346 | -0.101 |
| Proline | 0.423 | -0.118 | 0.081 | -0.467 | -0.342 | 0.122 | -0.135 | 0.039 | -0.208 | -0.417 | 0.105 | .743^*^ | -0.309 |
| Cystein | 0.495 | 0.047 | 0.374 | -0.246 | -0.367 | 0.161 | -0.241 | -0.282 | -0.651 | -0.661 | -0.350 | 0.618 | -0.670 |
| Tyrosine | 0.190 | 0.020 | 0.508 | 0.290 | -0.276 | 0.015 | 0.392 | 0.012 | -0.233 | -0.652 | -0.269 | 0.428 | -0.587 |
| Valine | 0.116 | -0.567 | 0.162 | -0.028 | -0.532 | 0.104 | 0.422 | .711^*^ | 0.268 | -0.039 | 0.260 | 0.437 | 0.016 |
| Methionine | 0.568 | -0.155 | 0.382 | -0.163 | -0.573 | -0.154 | -0.295 | -0.344 | -.729^*^ | -0.590 | -0.572 | .769^*^ | -.826^*^ |
| Lysin | 0.358 | -0.218 | -0.293 | -.802^*^ | -0.475 | 0.422 | -0.678 | -0.299 | -.769^*^ | -0.050 | -0.145 | 0.312 | -0.127 |
| Isoleusin | -0.209 | -0.497 | -0.137 | -0.065 | -0.305 | 0.134 | 0.523 | .715^*^ | 0.555 | 0.304 | 0.560 | 0.151 | 0.346 |
| Leusin | 0.072 | -0.510 | 0.160 | 0.028 | -0.439 | 0.092 | 0.485 | .745^*^ | 0.368 | -0.038 | 0.315 | 0.385 | 0.074 |
| Phenylalanine | 0.000 | 0.048 | 0.282 | 0.150 | -0.139 | 0.262 | 0.541 | 0.210 | 0.028 | -0.503 | 0.113 | 0.272 | -0.238 |
| TAA | 0.455 | -0.430 | 0.026 | -0.603 | -0.659 | 0.379 | -0.250 | 0.206 | -0.436 | -0.252 | 0.024 | 0.635 | -0.211 |
| Fat content | 0.316 | 0.106 | 0.605 | 0.244 | -0.156 | -0.311 | -0.097 | -0.309 | -0.337 | -0.431 | -0.474 | 0.419 | -.754^*^ |
| FI | -0.659 | -0.278 | -0.626 | -0.194 | 0.039 | 0.112 | 0.210 | 0.201 | 0.503 | .926^**^ | 0.612 | -0.460 | 0.639 |
| TPC | -0.075 | -0.096 | 0.671 | .898^**^ | -0.090 | -0.364 | 0.666 | 0.313 | 0.243 | -0.256 | -0.333 | -0.016 | -0.369 |

Suplementary Table 2. Pearson’s coefficient of correlations of amino acid content and the other chemical compounds. Values with * and ** were significant at confidence level 95% and 99%, respectively.

|  | Aspartic | Serine | Glutamic | Glycine | Histidine | Arginine | Threonine | Alanine | Proline | Cystein | Tyrosine | Valine | Methionine | Lysin | Isoleusin | Leusin | Phenylalanine | TAA |
| --- | --- | --- | --- | --- | --- | --- | --- | --- | --- | --- | --- | --- | --- | --- | --- | --- | --- | --- |
| Citric Acid | 0.660 | 0.297 | 0.527 | -0.006 | -0.026 | -0.110 | 0.688 | -0.413 | -0.104 | -0.478 | -0.092 | 0.619 | -0.386 | -0.424 | 0.706 | 0.655 | 0.053 | -0.006 |
| Acetic acid | -0.521 | 0.000 | -0.544 | 0.096 | 0.271 | -0.065 | -0.174 | -0.475 | -0.106 | -0.068 | 0.092 | -0.229 | -0.461 | -0.554 | -0.137 | -0.126 | 0.281 | -0.416 |
| Lactic acid | 0.299 | 0.125 | 0.284 | -0.206 | -0.271 | 0.213 | 0.237 | -0.095 | 0.069 | 0.112 | 0.000 | 0.084 | 0.283 | -0.040 | 0.024 | 0.043 | -0.201 | 0.043 |
| Glucose | 0.449 | .808^*^ | 0.436 | 0.345 | 0.574 | 0.360 | .837^**^ | -0.128 | 0.275 | 0.376 | 0.624 | 0.660 | 0.265 | -0.210 | 0.550 | 0.637 | 0.633 | 0.348 |
| Fructose | 0.446 | 0.563 | 0.430 | 0.501 | 0.497 | 0.240 | 0.524 | -0.449 | 0.312 | 0.000 | 0.272 | 0.629 | -0.314 | -0.574 | 0.597 | .715^*^ | 0.527 | 0.132 |
| Aspartic | 1 | 0.423 | .938^**^ | 0.579 | 0.220 | 0.086 | 0.634 | 0.236 | 0.614 | 0.107 | 0.149 | .884^**^ | 0.215 | 0.159 | .840^**^ | .857^**^ | 0.281 | 0.677 |
| Serine | 0.423 | 1 | 0.549 | 0.582 | .874^**^ | 0.305 | .786^*^ | -0.094 | 0.488 | 0.598 | .904^**^ | .735^*^ | 0.442 | -0.213 | 0.479 | .729^*^ | .910^**^ | 0.471 |
| Glutamic | .938^**^ | 0.549 | 1 | 0.627 | 0.364 | 0.236 | 0.558 | 0.327 | 0.669 | 0.314 | 0.316 | .852^**^ | 0.409 | 0.255 | 0.671 | .813^*^ | 0.371 | .770^*^ |
| Glycine | 0.579 | 0.582 | 0.627 | 1 | .750^*^ | 0.436 | 0.365 | 0.448 | .890^**^ | 0.640 | 0.516 | .735^*^ | 0.373 | 0.277 | 0.596 | .719^*^ | .720^*^ | .843^**^ |
| Histidine | 0.220 | .874^**^ | 0.364 | .750^*^ | 1 | 0.431 | 0.521 | 0.129 | 0.537 | 0.692 | .863^**^ | 0.608 | 0.380 | -0.022 | 0.370 | 0.604 | .958^**^ | 0.555 |
| Arginine | 0.086 | 0.305 | 0.236 | 0.436 | 0.431 | 1 | -0.093 | 0.414 | 0.276 | 0.516 | 0.177 | 0.155 | 0.171 | 0.356 | -0.037 | 0.121 | 0.219 | 0.503 |
| Threonine | 0.634 | .786^*^ | 0.558 | 0.365 | 0.521 | -0.093 | 1 | -0.280 | 0.300 | 0.127 | 0.586 | .834^**^ | 0.143 | -0.371 | .795^*^ | .839^**^ | 0.667 | 0.283 |
| Alanine | 0.236 | -0.094 | 0.327 | 0.448 | 0.129 | 0.414 | -0.280 | 1 | 0.467 | 0.493 | 0.003 | 0.107 | 0.540 | .982^**^ | -0.014 | 0.000 | -0.022 | .768^*^ |
| Proline | 0.614 | 0.488 | 0.669 | .890^**^ | 0.537 | 0.276 | 0.300 | 0.467 | 1 | .722^*^ | 0.502 | 0.633 | 0.619 | 0.330 | 0.470 | 0.598 | 0.575 | .825^*^ |
| Cystein | 0.107 | 0.598 | 0.314 | 0.640 | 0.692 | 0.516 | 0.127 | 0.493 | .722^*^ | 1 | .767^*^ | 0.269 | .845^**^ | 0.399 | -0.042 | 0.205 | 0.646 | 0.675 |
| Tyrosine | 0.149 | .904^**^ | 0.316 | 0.516 | .863^**^ | 0.177 | 0.586 | 0.003 | 0.502 | .767^*^ | 1 | 0.487 | 0.648 | -0.103 | 0.203 | 0.466 | .912^**^ | 0.411 |
| Valine | .884^**^ | .735^*^ | .852^**^ | .735^*^ | 0.608 | 0.155 | .834^**^ | 0.107 | 0.633 | 0.269 | 0.487 | 1 | 0.215 | -0.023 | .912^**^ | .991^**^ | 0.668 | 0.677 |
| Methionine | 0.215 | 0.442 | 0.409 | 0.373 | 0.380 | 0.171 | 0.143 | 0.540 | 0.619 | .845^**^ | 0.648 | 0.215 | 1 | 0.516 | -0.089 | 0.119 | 0.394 | 0.618 |
| Lysin | 0.159 | -0.213 | 0.255 | 0.277 | -0.022 | 0.356 | -0.371 | .982^**^ | 0.330 | 0.399 | -0.103 | -0.023 | 0.516 | 1 | -0.134 | -0.135 | -0.178 | 0.662 |
| Isoleusin | .840^**^ | 0.479 | 0.671 | 0.596 | 0.370 | -0.037 | .795^*^ | -0.014 | 0.470 | -0.042 | 0.203 | .912^**^ | -0.089 | -0.134 | 1 | .924^**^ | 0.472 | 0.477 |
| Leusin | .857^**^ | .729^*^ | .813^*^ | .719^*^ | 0.604 | 0.121 | .839^**^ | 0.000 | 0.598 | 0.205 | 0.466 | .991^**^ | 0.119 | -0.135 | .924^**^ | 1 | 0.674 | 0.598 |
| Phenylalanine | 0.281 | .910^**^ | 0.371 | .720^*^ | .958^**^ | 0.219 | 0.667 | -0.022 | 0.575 | 0.646 | .912^**^ | 0.668 | 0.394 | -0.178 | 0.472 | 0.674 | 1 | 0.477 |
| TAA | 0.677 | 0.471 | .770^*^ | .843^**^ | 0.555 | 0.503 | 0.283 | .768^*^ | .825^*^ | 0.675 | 0.411 | 0.677 | 0.618 | 0.662 | 0.477 | 0.598 | 0.477 | 1 |
| Fat content | -0.137 | 0.510 | -0.018 | 0.205 | 0.409 | 0.338 | 0.189 | -0.047 | 0.391 | .755^*^ | 0.667 | 0.014 | 0.620 | -0.096 | -0.194 | -0.013 | 0.468 | 0.154 |
| FI | 0.253 | -0.359 | -0.069 | -0.132 | -0.403 | -0.279 | 0.210 | 0.029 | -0.180 | -0.524 | -0.491 | 0.131 | -0.418 | 0.038 | 0.480 | 0.119 | -0.313 | -0.072 |
| TPC | -0.065 | 0.699 | 0.013 | -0.137 | 0.447 | -0.082 | 0.641 | -0.654 | -0.201 | 0.099 | 0.642 | 0.239 | 0.059 | -0.673 | 0.099 | 0.276 | 0.540 | -0.273 |

Suplementary Table 3. Pearson’s coefficient of correlations of organic acid, sugars, fat and total phenolic content and fermentation index. Values with * and ** were significant at confidence level 95% and 99%, respectively.

|  | Citric Acid | Acetic acid | Lactic acid | Glucose | Fructose | Fat content | FI | TPC |
| --- | --- | --- | --- | --- | --- | --- | --- | --- |
| Citric Acid | 1 | -0.289 | 0.316 | 0.483 | 0.570 | -0.350 | 0.380 | 0.359 |
| Acetic acid | -0.289 | 1 | -0.610 | -0.235 | 0.403 | 0.048 | -0.228 | 0.156 |
| Lactic acid | 0.316 | -0.610 | 1 | 0.490 | -0.038 | 0.485 | 0.098 | 0.189 |
| Glucose | 0.483 | -0.235 | 0.490 | 1 | 0.413 | 0.512 | 0.109 | 0.632 |
| Fructose | 0.570 | 0.403 | -0.038 | 0.413 | 1 | 0.027 | -0.157 | 0.377 |
| Fat content | -0.350 | 0.048 | 0.485 | 0.512 | 0.027 | 1 | -0.410 | 0.412 |
| FI | 0.380 | -0.228 | 0.098 | 0.109 | -0.157 | -0.410 | 1 | -0.309 |
| TPC | 0.359 | 0.156 | 0.189 | 0.632 | 0.377 | 0.412 | -0.309 | 1 |
